# Oesophageal squamous cell carcinoma–associated IL‐33 rewires macrophage polarization towards M2 via activating ornithine decarboxylase

**DOI:** 10.1111/cpr.12960

**Published:** 2020-12-10

**Authors:** Shijie Mai, Le Liu, Jianjun Jiang, Pengfei Ren, Dingwei Diao, Haofei Wang, Kaican Cai

**Affiliations:** ^1^ Department of Thoracic Surgery Nanfang Hospital Southern Medical University Guangzhou China; ^2^ Department of Gastroenterology Nanfang Hospital Southern Medical University Guangzhou China

**Keywords:** IL‐33, macrophage, oesophageal squamous cell carcinoma, ornithine decarboxylase

## Abstract

**Background:**

The tumour microenvironment primarily constitutes macrophages in the form of an immunosuppressive M2 phenotype, which promotes tumour growth. Thus, the development of methodologies to rewire M2‐like tumour‐associated macrophages (TAMs) into the M1 phenotype, which inhibits tumour growth, might be a critical advancement in cancer immunotherapy research.

**Methods:**

The expressions of IL‐33 and indicators related to macrophage polarization in oesophageal squamous cell carcinoma (ESCC) tissues and peripheral blood mononuclear cell (PBMC)–derived macrophages were determined. Inhibition of ornithine decarboxylase (ODC) with small interfering RNA was used to analyse the phenotype of macrophage polarization and polyamine secretory signals. CCK‐8, wound‐healing and Transwell assays were used to detect the proliferation and migration of ECA109 cells in vitro. The tumour xenograft assay in nude mice was used to examine the role of IL‐33 in ESCC development in vivo.

**Results:**

This study showed the substantially elevated IL‐33 expression in ESCC tissues compared with the normal tissues. Additionally, enhanced infiltration of M2‐like macrophages into the ESCC tumour tissue was also observed. We observed a strong correlation between the IL‐33 levels and the infiltration of M2‐like macrophages in ESCC tumours locally. Mechanistically, IL‐33 induces M2‐like macrophage polarization by activating ODC, a key enzyme that catalyses the synthesis of polyamines. Inhibition of ODC suppressed M2‐like macrophage polarization. Finally, in vivo, we confirmed that IL‐33 promotes tumour progression.

**Conclusions:**

This study revealed an oncogenic role of IL‐33 by actively inducing M2‐like macrophage differentiation; thus, contributing to the formation of an immunosuppressive ESCC tumour microenvironment. Thus, IL‐33 could act as a novel target for cancer immunotherapies.

## Graphical abstract

M2 macrophage infiltration and IL‐33 production are enhanced with close correlation in ESCC. (A) Representative images of IL‐33^+^ cell, CD206^+^ cell and CD68^+^ cell in non‐tumour and tumour tissue (scale bar = 50 μm). (B) Western blot analysis of IL‐33 and CD206 expression in non‐tumour and tumour tissues, and GAPDH was used as a reference control. (C) IL‐33, CD206 and CD68 were measured by RT‐PCR in non‐tumour and tumour tissues; the correlation of CD206 and IL‐33 in ESCC tissues; N = 20, *R* ^2^ = .54, *P* < .01. (D) M1 (CD68^+ ^CCR2^+^) and M2 (CD206^+ ^CX3CR1^+^) populations in peripheral blood from those with ESCC (n = 48) and healthy controls (n = 33), as measured by flow cytometry. The difference in M1/M2 ratio between two groups was analyzed. (E) The population of M2‐like monocytes subset in peripheral blood between ESCC patients and healthy controls showed significant difference instead of M1‐like monocytes. (F) ROC curve was used to analyse to assess the diagnostic value in ESCC. *P < .05; **P < .01; ***P < .001. Bars indicate mean and SEM of triplicate experiments and show a representative experiment of at least 3 independent experiments performed for each panel.

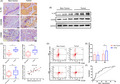

## 1 INTRODUCTION

In our immune system, macrophages constitute a major heterogeneous cellular population, which protects our body against several types of pathogenic infections, such as bacterial, fungal and viral. The macrophages located in the tumour microenvironment (TME) are classified as tumour‐associated macrophages (TAMs).^1^ The macrophage colony‐stimulating factor (M‐CSF), the C‐C motif chemokine ligand 2 (CCL2) and the vascular endothelial growth factor (VEGF) specifically recruit circulating monocytes, which are eventually transformed into TAMs.^2^ The differentiation of macrophages into the M1 type (classically activated) occurs under the influence of LPS or IFN‐γ,^3^, ^4^ and into the M2 type (alternatively activated) through IL‐4 and IL‐13.^3^ The M1 type macrophages engulf and destroy pathogenic microbes that cause viral, bacterial or fungal diseases. They also produce pro‐inflammatory cytokines, such as IL‐12 and TNF‐α.^5^ On the contrary, M2‐like macrophages produce anti‐inflammatory cytokines, such as transforming growth factor‐β (TGF‐β) and IL‐10, which promote the inhibition of these adaptive Th1 immune responses and the resulting inflammation.^6^, ^7^ Additionally, they exhibit an IL‐12^low ^IL‐10^high^ phenotype and are involved in angiogenesis, tissue remodelling, parasitic infections and tumorigenesis.^8^, ^9^, ^10^ However, there are limited reports on the mechanism of M2‐like macrophage differentiation and its role in human oesophageal squamous cell carcinoma (ESCC) is unclear.

IL‐33, a pro‐inflammatory cytokine from the IL‐1 family, regulates the host immune response and promotes tumour growth.^11^, ^12^ Several studies have revealed that elevated IL‐33 levels are probable prognostic markers and correspond to poor prognosis in many types of cancers.^13^, ^14^, ^15^ Currently, research data indicate an association between IL‐33 and the pathogenesis of ESCC.^16^ Elevated IL‐33 levels in ESCC are related to the invasion of Treg cells in the tumour. Moreover, the Treg cells express ST2, suggesting that IL‐33 probably regulates the physiological activity of Treg cells through ST2.^17^, ^18^

Previous studies have indicated that IL‐13/IL‐4Rα signalling pathway is involved in the amplification and polarization of the alveolar and bone marrow–derived macrophages, resulting in airway inflammation.^19^ Additionally, IL‐33 is also known to regulate macrophage polarization. In a murine model, IL‐33 improved CVB3‐induced viral myocarditis by inducing macrophage polarization to the M2 type.^20^ IL‐33 promoted IL‐10 expression in macrophages through activating ERK 1/2 and STAT3, which subsequently promoted IL‐10 transcription and thus contributed to the beneficial effects of IL‐33 on macrophages.^21^ Similarly, in the colon cancer TME, IL‐33 promoted tumorigenesis by inducing macrophage polarization.^22^ However, there are no reports on the in‐depth investigation of the role of IL‐33 in macrophage polarization to M2 type in ESCC, whether it promotes further tumorigenesis and its mechanism of action.

Here, we found that in patients with ESCC, the tumour tissue samples had elevated levels of IL‐33 compared with non‐tumour tissue samples. Additionally, an elevated count of CD206^+^ M2 type macrophages, which infiltrated the ESCC tumours, was observed, and this increase had a positive correlation with the secretion of IL‐33. In vitro studies revealed that IL‐33 favoured macrophage polarization towards the M2 phenotype, consistent with the expression of CD206^+^ by activating ornithine decarboxylase (ODC). Inhibition of ODC using small interfering RNA (siRNA) suppressed M2‐like macrophages polarization. The supernatants from IL‐33–induced M2‐like macrophages promoted ESCC cell progression. Thus, these results describe the relevance of the IL‐33/macrophage axis in the clinical treatment of ESCC.

## 2 MATERIALS AND METHODS

### 2.1 Patients and tissue samples

Patients who were admitted to the Southern Hospital of Southern Medical University and who had not received chemotherapy/radiotherapy underwent surgical resection to provide tumorous and non‐tumorous (minimum 5 cm from the tumour) oesophageal tissue samples. Histopathological studies confirmed the absence of cancer cell infiltration in the non‐tumour tissue samples. All patients provided written informed consent before study initiation. The TNM staging system (the International Union Against Cancer, 8th edition) was used to identify the clinicopathological stages of tumours. The Ethics Committee of the Southern Hospital of Southern Medical University sanctioned this study.

### 2.2 Immunohistochemistry

The ESCC tissue samples were embedded in paraffin, and 4 µm sections were obtained. Next, these sections were treated with normal goat serum for 30 minutes at 37°C, followed by overnight incubation at 4°C with the following primary antibodies: rabbit anti‐CD206, rabbit anti‐IL‐33 or rabbit anti‐CD68 antibodies (Affinity, USA). After washing, these sections were incubated for 30 minutes at 37°C with horseradish peroxidase (HRP)–conjugated anti‐rabbit secondary antibodies (Affinity, USA). Two independent and experienced histopathologists who were blinded to clinical patient data reviewed each section. We identified five fields in each section and counted brown granule‐containing cells at 400× magnification for each field using average values.

### 2.3 Flow cytometry analysis

We collected blood samples from both healthy subjects and patients with ESCC, followed by isolation of human peripheral blood mononuclear cells (PBMCs) using Ficoll‐Hypaque density gradient centrifugation (GE Healthcare, USA). We isolated CD14^+^ monocytes from PBMCs using the human CD14 Positive Selection Kit (Stem Cell Technology, Vancouver, Canada) on the basis of the manufacturer's instructions. The purity of the monocytes was detected by flow cytometry. 100 ng/mL M‐CSF (PeproTech, NJ, USA) was used to induce monocytes differentiate into adherent macrophages in 6‐well plates (NEST Biotechnology, Wuxi, China) for 5 days at 37°C. Then, the identification of M1‐like or M2‐like macrophages was done by staining the cells FITC anti‐CD68 mAb and PE anti‐CCR2 mAb or APC anti‐CX3CR1 and PE anti‐CD163 antibodies (BD Biosciences). The percentage of the total macrophage gate revealed the relative frequency of the specific macrophage subset. A Beckman Coulter Cyan Flow Cytometer was used for cell phenotyping post‐appropriate compensations. FlowJo v8.5.2 was used for the analysis of the flow cytometric data (Tree Star, Inc, OR).

### 2.4 Cell culture and M2 macrophage induction

Fresh blood samples from healthy donors were collected, and PBMCs were isolated by density gradient centrifugation using Ficoll‐Hypaque (GE Healthcare, NJ, USA). Isolation of monocytes and induction into M0 macrophages were done using the above procedures. Next, human recombinant IL‐33 (PeproTech, New Jersey, USA) was used to induce M0 macrophages to differentiate into M2 macrophages for 24 hours. All cells were cultured at 37°C in a humidified incubator with 5% CO_2_.

### 2.5 Immunofluorescence

We induced the M0/M2 macrophages, at a concentration of 5 × 10^5^ cells/well, using the method described in the previous section. After washing with PBS, the cells were treated with 5% goat serum in PBS for 30 minutes, followed by incubation with rabbit anti‐human ODC and arginase 1 (ARG1) primary antibody (Affinity, USA) diluted in the antibody dilution. This antigen‐antibody complex was coupled with a tetramethylrhodamine‐conjugated goat anti‐rabbit secondary antibody (Zhongshan Biotechnology). Finally, the PBS‐washed cells were visualized using a microscope (Olympus, Japan).

### 2.6 siRNA transfection of macrophages

We induced the M0 macrophages, at a concentration of 5 × 10^5^ cells/well, using the method described in the previous section. Next, these macrophages were transfected for 6 hours with either non‐silencing control or ODC‐targeting siRNA (40 pmol/mL), following the manufacturer's protocol (GenePharma, Shanghai, China). Post‐transfection, the cells were grown for another 24 hours in fresh RPMI‐1640 medium containing FBS (10%), followed by a 24 hours treatment with IL‐33 (20 ng/mL) to induce their differentiation into M2 macrophages.

### 2.7 Quantitative RT‐PCR

The TRIzol reagent (Vazyme, Nanjing, China) was used for total RNA extraction from the cultured cells, following the manufacturer's protocol. The cDNA was obtained by reverse‐transcribing the isolated RNA (500 ng/10 µL) using a reverse transcription kit (Vazyme, Nanjing, China) and was diluted in 10 µL of nuclease‐free water. A Roche LC480 System was used to perform Real‐time PCR by mixing SYBR Green Master Mix (Vazyme, Nanjing, China) with 2 µL cDNA using these forward and reverse primers (GenePharma, China): human IL‐12p35 (forward 5′‐CCA GAA GGC CAG ACA AAC‐3′, reverse 5′‐CCA GGC AAC TCC CAT TAG‐3′); CD68 (forward 5′‐GCC CTG GTG CTT ATT GCT‐3′, reverse 5′‐TTT GAG CCA GTT GCG TGT‐3′); CD206 (forward 5′‐CGA GGA AGAGGT TCG GTT CACC‐3′, reverse 5′‐GCA ATC CCG GTTCTC ATG GC‐3′); IL‐10 (forward 5′‐GCT GTC ATCGAT TTC TTC CC‐3′, reverse 5′‐CTC ATG GCT TTG TAGATG CCT‐3′); TGF‐β (forward 5′‐AAC TACTGC TTC AGC TCC AC‐3′, reverse 5′‐TGT GTC CAG GCTCCA AAT GTA‐3′); IL‐33 (forward 5′‐AGA GAA ACC ACC AAA AGG C‐3′, reverse 5′‐ATA CCA AAG GCA AAG CAC TC‐3′); and GADPH (forward 5′‐CCT TCC GTG TCC CCA CT‐3′, reverse 5′‐GCC TGC TTC ACC ACC TTC‐3′). We used GADPH as the reference and the 2^−△△CT^ method to determine the relative expression of the target mRNAs.

### 2.8 Western blot analysis

We used the RIPA Lysis Buffer (Fudebio, Hangzhou, China) containing phosphatase/protease inhibitors (Fudebio, Hangzhou, China) to extract total protein from the cultured cells following the manufacturer’s method. SDS‐polyacrylamide gel electrophoresis (10%; SDS‐PAGE) was performed to separate the protein samples (20 µg), which were then transferred to Millipore polyvinylidene difluoride (PVDF) membranes. These PVDF membranes were kept in overnight incubation at 4°C with anti‐CD206, anti‐ODC, anti‐IL‐33, anti‐iNOS, anti‐CD68, anti‐ARG1 and anti‐GADPH primary antibodies (Affinity, USA), followed by incubation at room temperature for 60 minutes with horseradish peroxidase (HRP)–conjugated secondary antibodies (Affinity, USA) that were diluted to 1:5000 in 5% skim milk. After washing with TBST, the membranes were treated for 1 minutes with SuperSignal™ West Dura Extended Duration Substrate (Fudebio, Hangzhou, China) and were visualized through chemiluminescence.

### 2.9 Collection of supernatants

We induced the M0 macrophages using the method described in the previous section. The resultant M0 macrophages were treated with IL‐33 (20 ng/mL) for a period of 24 hours to induce their differentiation into M2‐like macrophages. Next, fresh RPMI‐1640 medium (400 µL/well) containing FBS (10%) was added to both M0 and M2 macrophages, and we harvested the cell‐free supernatants from M0‐ or M2‐like macrophages post another 24 hours culture.

### 2.10 Determination of polyamines by high‐performance liquid chromatography (HPLC)

1.0 mL of macrophage supernatant was collected, followed by the addition of 1 mL of 2 mol/L NaOH solution and 5 µL of benzoyl chloride, followed by vortex mixing, and then allowed to stand for 20 minutes. 2 mL of saturated sodium chloride solution and 2 mL of chloroform were added, followed by vortex mixing for 1 minutes, then centrifugation at 2200 *g* for 10 minutes, and finally, the lower organic phase was collected and blown dry with nitrogen. The resulting white solid was dissolved in 0.5 mL chromatographic methanol, then placed in a 50°C water bath for 8 hours, then 0.2 mL 2 mol/L NaOH solution and 2 mL chloroform were added, followed by vortex mixing, followed by centrifugation at 2200 *g* for 10 minutes, and finally the lower organic phase was collected and blown dry with nitrogen. The resulting white solid was dissolved with 0.5 mL chromatographic methanol, and filtered with 0.22 µm organic microporous membrane. Chromatographic conditions are as follows: detection wavelength: 234 nm; column temperature: 25°C; mobile phase: 55% methanol‐45% water; flow rate: 1.0 mL/min; and injection volume: 20 µL.

### 2.11 Cell proliferation and migration analysis

We co‐cultured the ECA‐109 cell line (5 × 10^3^ cells/well) with the supernatants of M2‐like macrophages in 96‐well plates (NEST Biotechnology, Wuxi, China) to study cellular proliferation. We used a Cell Counting Kit‐8 (CCK‐8; Fudebio, Hangzhou, China) to detect their absorbance values at 12, 24, 36 and 48 hours following the manufacturer's protocol. A phase‐contrast microscope was used to capture images of the wound‐healing assay at 0 and 48 hours, and measure the cell migration distance. The L_0_ h‐L_48_ h was defined as the length of wound healing. The wound‐healing assay was performed in triplicates. The ECA‐109 cells (1 × 10^5^ cells/well) in serum‐free RPMI‐1640 medium were added to a 24‐well plate containing Transwell inserts (Corning, USA) to determine cellular migration. Next, the supernatants of the M2‐like macrophage (600 µL) were placed in the lower chamber and incubated at 37°C. Twenty‐four hours later, we stained the cells in the lower chamber, counted the cells in five different fields of vision and imaged them using a microscope (Olympus, Japan).

### 2.12 Animal experiments

We procured female BALB/c nude mice (6‐8 weeks old) from Guangdong Medical Laboratory Animal Center (Guangzhou, China) to perform the xenograft experiments. The ECA‐109 cells (5 × 10^6^) were subcutaneously injected into the mice at the left fourth mammary fat pat to create the ESCC tumour model. Seven days later, the mice were randomly assigned to two different groups and subcutaneously injected with either IL‐33 (200 ng/mouse) or PBS around solid tumour site every alternate day. The mice were injected 11 times during the whole experimental period. Next, we measured the tumour size and calculated the tumour volume using the following equation: Volume = (length × width^2^)/2. On day 21, mice were euthanized, followed by H&E staining analysis. During the survival experiment, we injected the mice every alternate day for 33 days, after which they were injected once every three days. We monitored the mice daily, and all animal experiments are carried out subject to the NIH’s Guide for the Care and Use of Laboratory Animals.

### 2.13 Statistical analysis

All statistical analyses were done using spss v20.0. The data were represented as mean ± SEM (standard error of the mean). Student's *t* test was performed to determine the statistical significance of the differences between the two groups. The multi‐group data analysis was done using ANOVA. The Pearson correlation analysis or the linear regression analysis was used to evaluate the correlations between parameters. Data analysis was done using two‐tailed tests; *P* < .05 was regarded as statistically significant unless stated otherwise.

## 3 RESULTS

### 3.1 ESCC progression is strongly correlated with M2‐like macrophage infiltration and IL‐33 secretion

**Figure 1:**
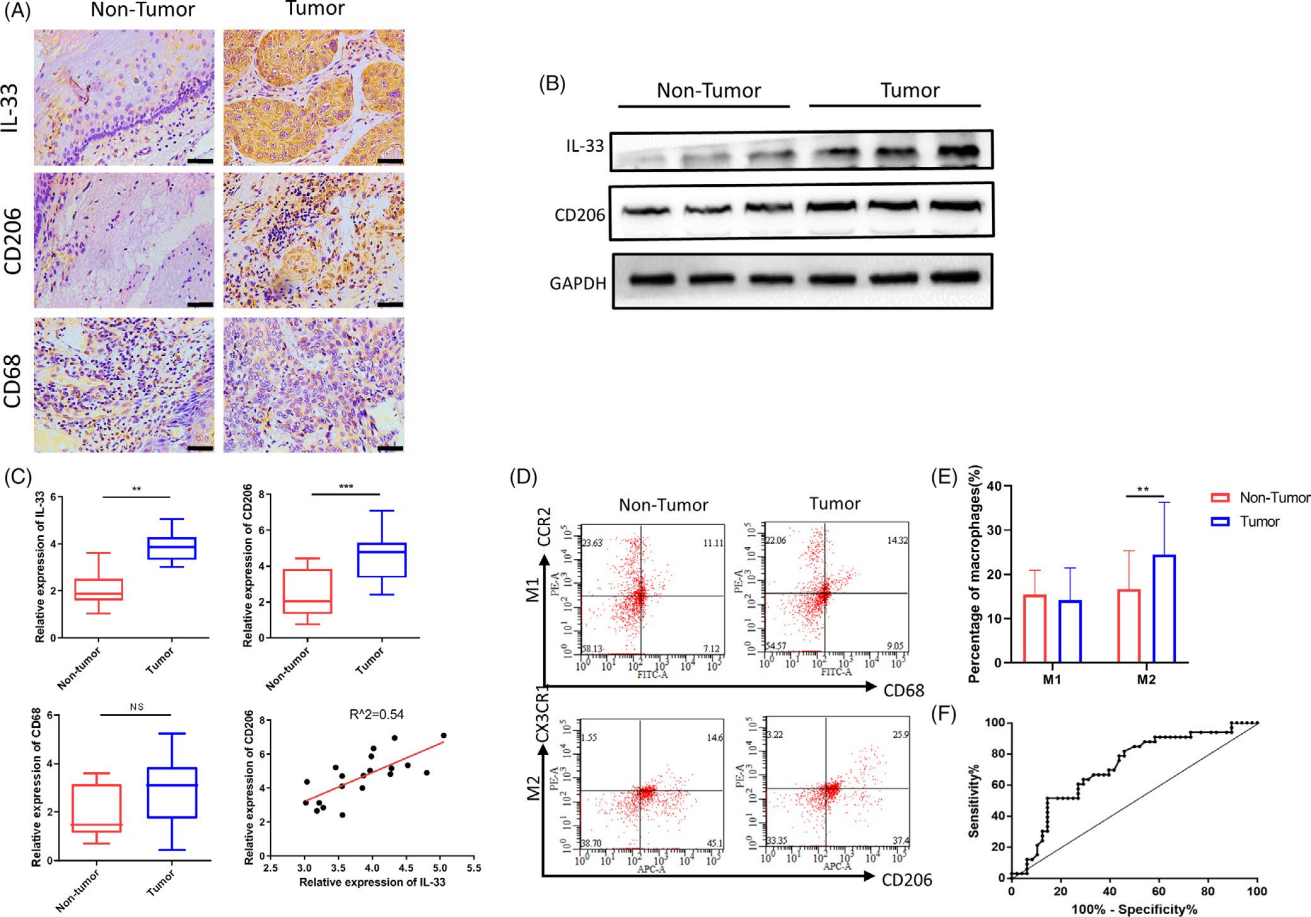
M2 macrophage infiltration and IL‐33 production are enhanced with close correlation in oesophageal squamous cell carcinoma (ESCC). A, Representative images of IL‐33^+^ cell, CD206^+^ cell and CD68^+^ cell in non‐tumour and tumour tissue (scale bar = 50 μm). B, Western blot analysis of IL‐33 and CD206 expression in non‐tumour and tumour tissues, and GAPDH was used as a reference control. C, IL‐33, CD206 and CD68 were measured by RT‐PCR in non‐tumour and tumour tissues; the correlation of CD206 and IL‐33 in ESCC tissues; N = 20, *R* ^2^ = .54, *P* < .01. D, M1 (CD68^+^ CCR2^+^) and M2 (CD206^+^ CX3CR1^+^) populations in peripheral blood from those with ESCC (n = 48) and healthy controls (n = 33), as measured by flow cytometry. The difference in M1/M2 ratio between two groups was analysed. E, The population of M2‐like macrophages subset in peripheral blood between ESCC patients and healthy controls showed significant difference instead of M1‐like macrophages. F, ROC curve was used to analyse to assess the diagnostic value in ESCC. **P < .01; ***P < .001. Bars indicate mean and SEM of triplicate experiments and show a representative experiment of at least 3 independent experiments performed for each panel

We performed immunohistochemical studies to assess the IL‐33, CD68 and CD206 levels in the tumour and non‐tumour tissue samples from patients with ESCC. IL‐33 is mainly located in the cytoplasm of ESCC cells, while CD68 and CD206 are mainly found in the tumour stroma. Substantially elevated levels of IL‐33 and CD206 but not CD68 were observed in the tumour tissue samples than the non‐tumour tissue samples (Figure 1A). We performed WB to determine IL‐33 levels to verify the previous results and found considerably elevated secretion of IL‐33 in tumour tissues compared with the non‐tumour tissues (Figure 1B). Moreover, we found a positive correlation between IL‐33 expression and the number of CD206^+^ macrophages (Figure 1C). However, CD68^+^ macrophages were not correlated with IL‐33 production. Table 1 presents the baseline clinical and pathological characteristics. Both IL‐33 and CD206 were related to the TNM stage and lymph node status. At a high TNM stage (stage III) and lymph node metastasis (N1‐3), the expressions of IL‐33 and CD206 were significantly upregulated (*P* < .05). But we found no correlation between IL‐33/CD206 and the T stage, degree of differentiation, age and gender. Flow cytometric analysis was done to detect the macrophage subsets in patients with ESCC and in healthy donors: CD68 and CCR2 double positive were used to label the M1‐like macrophages, and CD163 and CX3CR1 double positive were used to label the M2‐like macrophages. We found no significant difference in the percentage of M1‐like macrophages between patients with ESCC and healthy donors; however, a significant increase in the percentage of M2‐like macrophages was observed in patients with ESCC (Figure 1D‐E). ROC curve was used to evaluate the role of M2‐like macrophages in the auxiliary diagnosis of ESCC. The results showed that the area under the ROC curve (AUC) was 0.7102 (95% CI: 59.58%‐82.46%, *P* = .0014), which indicated that the percentage of M2‐like macrophages could be used as an indicator for the auxiliary diagnosis of ESCC (Figure 1F).

**Table 1:** Correlative analysis of expression levels of IL‐33 and CD206 with clinicopathological factors

Features	n	IL‐33				CD206			
		High	Low	χ^2^	*P*	High	Low	χ^2^	*P*
Age (y)									
<65	55	34	21	0.169	.681	18	37	0.149	.7
≥65	28	16	12			8	20		
Gender									
Male	66	39	27	0.178	.673	19	47	0.964	.326
Female	17	11	6			7	10		
G stage									
G1	19	12	7	0.088	.767	3	16	2.765	.096
G2‐3	64	38	26			23	41		
Location									
Low	40	26	14	0.73	.393	14	26	0.485	.486
Middle to upper	43	24	19			12	31		
TNM stage									
Ⅰ‐Ⅱ stage	43	17	26	15.972	.001	8	35	6.711	.01
Ⅲ stage	40	33	7			18	22		
T stage									
1‐2	21	12	9	0.113	.737	6	15	0.099	.753
3‐4	62	38	24			20	42		
N stage									
N0	47	22	25	8.163	.004	10	37	5.086	.024
N1‐3	36	28	8			16	20		

### 3.2 IL‐33 skews M2‐like macrophage polarization

**Figure 2:**
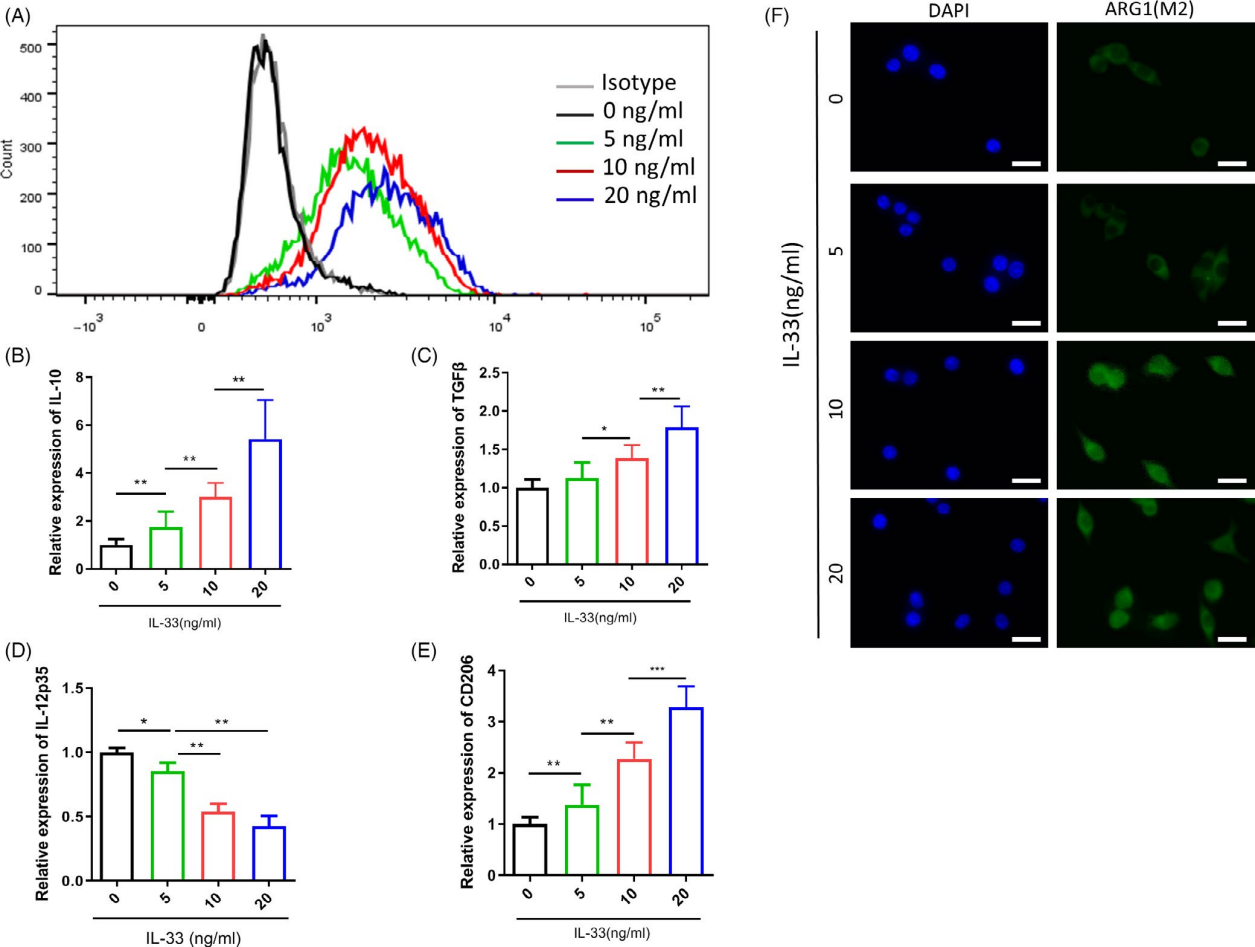
IL‐33 skews M2 macrophage polarization. A, Expression of CD206 determined by flow cytometry in IL‐33–induced macrophage increased with increasing IL‐33 concentration. B‐E), IL‐10, TGF‐β, IL‐12p35 and CD206 determined by qPCR in IL‐33–induced macrophage increased with increasing IL‐33 concentration. F, Immunofluorescence staining for IL‐33–induced M2 macrophages (scale bar = 20 μm). The green signal represents the staining of activated ornithine decarboxylase, and the blue signal represents the DAPI‐stained nuclei. *P < .05; **P < .01; ***P < .001. Bars indicate mean and SEM of triplicate experiments and show a representative experiment of at least 3 independent experiments performed for each panel

Based on the IL‐33 levels in this study and their association with the enhanced infiltration of local M2 macrophage in ESCC tumours, we investigated whether IL‐33 could induce the M0‐to‐M2 macrophage differentiation by treating the M‐CSF–induced PBMC‐derived macrophages with IL‐33. Post‐induction, the levels of CD206 were detected in the macrophages via flow cytometry. The results showed that IL‐33 significantly enhanced the CD206 expression in macrophages (Figure 2A). Also, RT‐PCR revealed that IL‐33 induction upregulated the mRNA expression of the M2 macrophage markers, such as TGF‐β, IL‐10 and CD206 (Figure 2B‐E). These results were further substantiated by the immunofluorescence analysis (Figure 2F). Thus, IL‐33 induced the M0 to M2 macrophage differentiation.

### 3.3 IL‐33 induces M2‐like macrophage ODC activation

**Figure 3:**
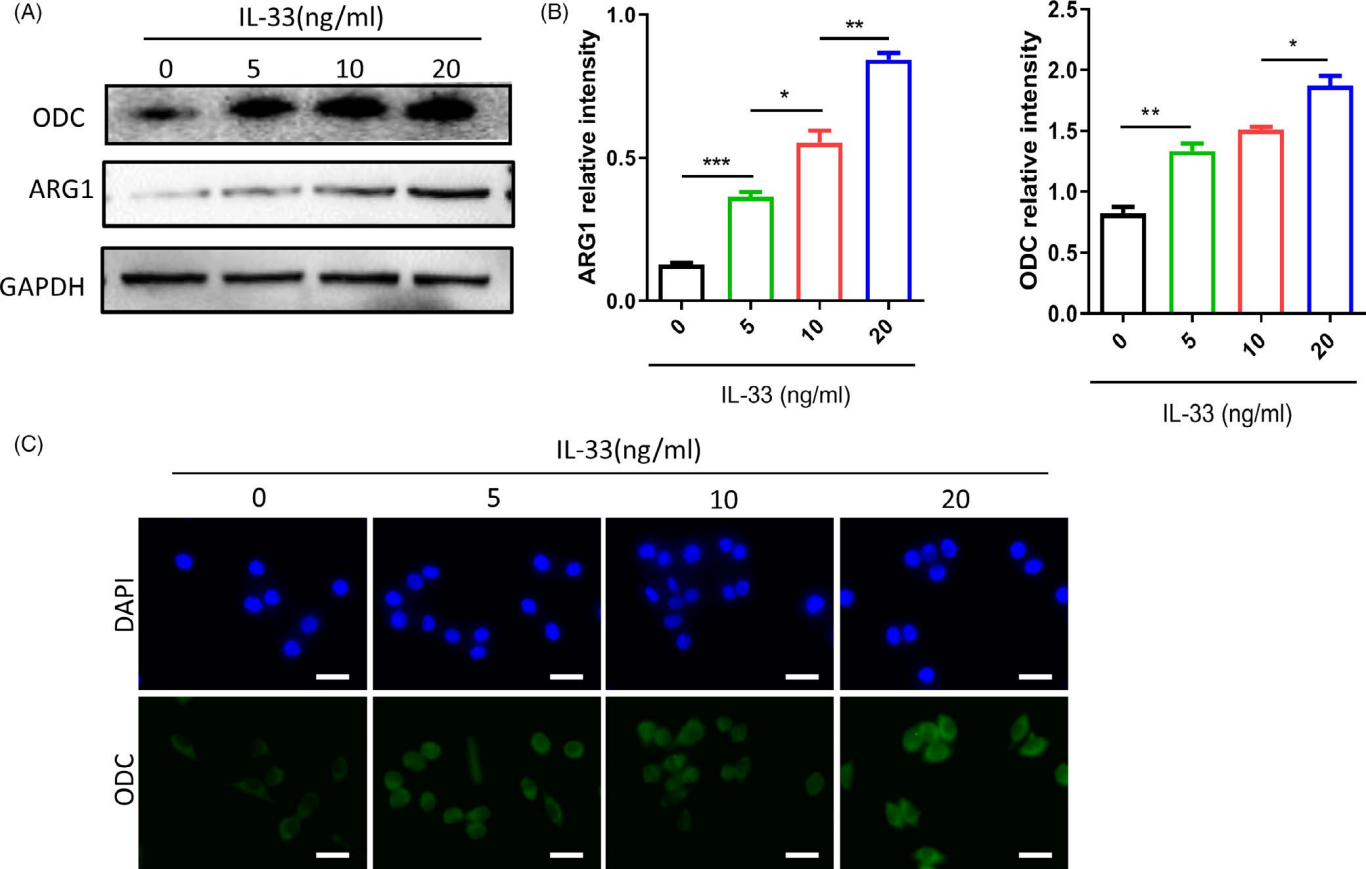
IL‐33 induces macrophage ornithine decarboxylase (ODC) activation. A, ODC and arginase 1 (ARG1) protein levels were determined by Western blot, and GAPDH was used as a reference control. B, Relative intensity of ODC and ARG1 protein. C, Immunofluorescence staining for IL‐33–induced M2 macrophages. The green signal represents the staining of activated ODC, and the blue signal represents the DAPI‐stained nuclei (scale bar = 20 μm). *P < .05; **P < .01; ***P < .001. Bars indicate mean and SEM of triplicate experiments and show a representative experiment of at least 3 independent experiments performed for each panel

Polyamine biosynthesis begins with ornithine and methionine. Ornithine decarboxylase (ODC) is the first rate‐limiting enzyme for polyamine synthesis. In mammals, ornithine is the product of the urea cycle, and ornithine can be transformed into putrescine under the action of ODC. Expression of ODC is strictly regulated at all stages of transcription, translation and post‐translation. Given that ODC high expression is closely related to tumorigenesis and development, anti‐tumour therapy studies with ODC as a molecular target have received high attention. The results suggested the involvement of ODC activation. Next, the IL‐33–induced M2‐type differentiation test was repeated, which showed the upregulation of the protein levels of ODC and ARG1 (aM2 marker), supported by WB (Figure 3A‐B) and the immunofluorescence experiments (Figure 3C).

### 3.4 IL‐33–induced M2‐like macrophage differentiation occurs via ODC activation

**Figure 4:**
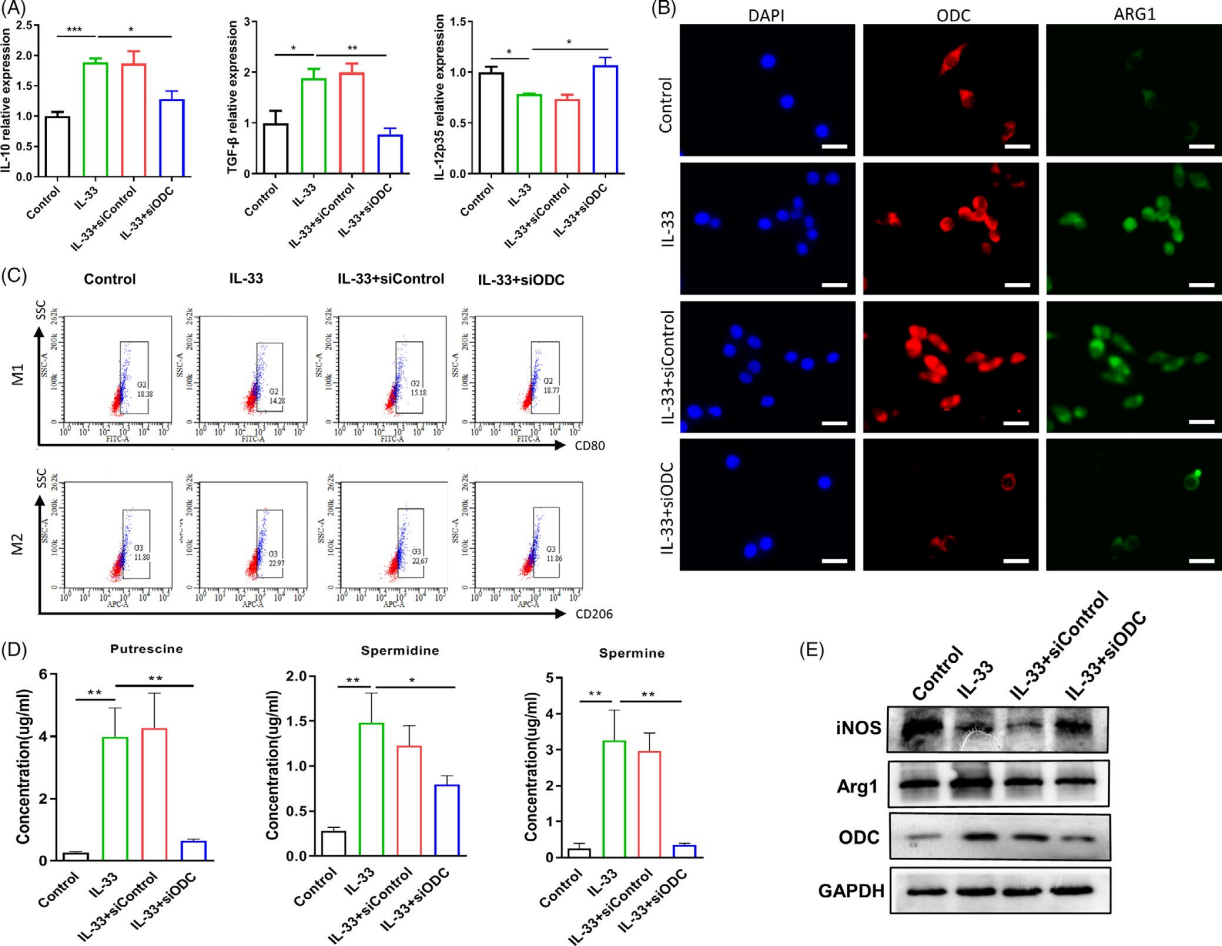
IL‐33 promotes M2 macrophage polarization via ornithine decarboxylase (ODC) activation. A, IL‐10, TGF‐β and IL‐12p35 protein levels were determined by Western blot in IL‐33–induced M2 macrophage with or without knocking down ODC. B, Immunofluorescence staining for IL‐33–induced M2 macrophages with or without knocking down ODC. The red signal represents the staining of ODC, and the green signal represents the staining of ARG1 (scale bar = 20 μm). C, The expression of CD80 (M1) and CD206 (M2) by flow cytometry in IL‐33–induced macrophages with or without knocking down ODC. D, Determination of polyamines in IL‐33–induced M2 macrophage supernatants with or without knocking down ODC by HPLC. E, iNOS and ARG1 protein levels were determined by Western blot in IL‐33–induced M2 macrophage with or without knocking down ODC. *P < .05; **P < .01; ***P < .001. Bars indicate mean and SEM of triplicate experiments and show a representative experiment of at least 3 independent experiments performed for each panel

Here, we treated cells with ODC‐targeting siRNA, followed by the repetition of the IL‐33–induced M2‐type macrophage polarization test to understand the regulatory role of ODC activation in the IL‐33 induced M2‐type macrophage polarization. We found substantially downregulated levels of IL‐10 and TGF‐β while upregulated levels of IL‐12p35 in the ODC siRNA group than the control siRNA group (Figure 4A). Thus, these results indicated that IL‐33 induced M2 macrophage differentiation with the IL‐10^high^TGF‐β^high^IL‐12_p35_^low^ phenotype through ODC activation. The inferences from immunofluorescence (Figure 4B), flow cytometry (Figure 4C) and WB (Figure 4E) also verified these results. We further used HPLC to detect the content of polyamines (putrescine, spermidine and spermine), downstream of ODC, in the supernatant of induced macrophages, and found increased levels of putrescine, spermidine and spermine in the supernatants of IL‐33–induced M2 macrophages and decreased levels in the ODC siRNA group (Figure 4D).

### 3.5 IL‐33–induced M2‐like macrophage supernatants support the migration of ESCC cells

**Figure 5:**
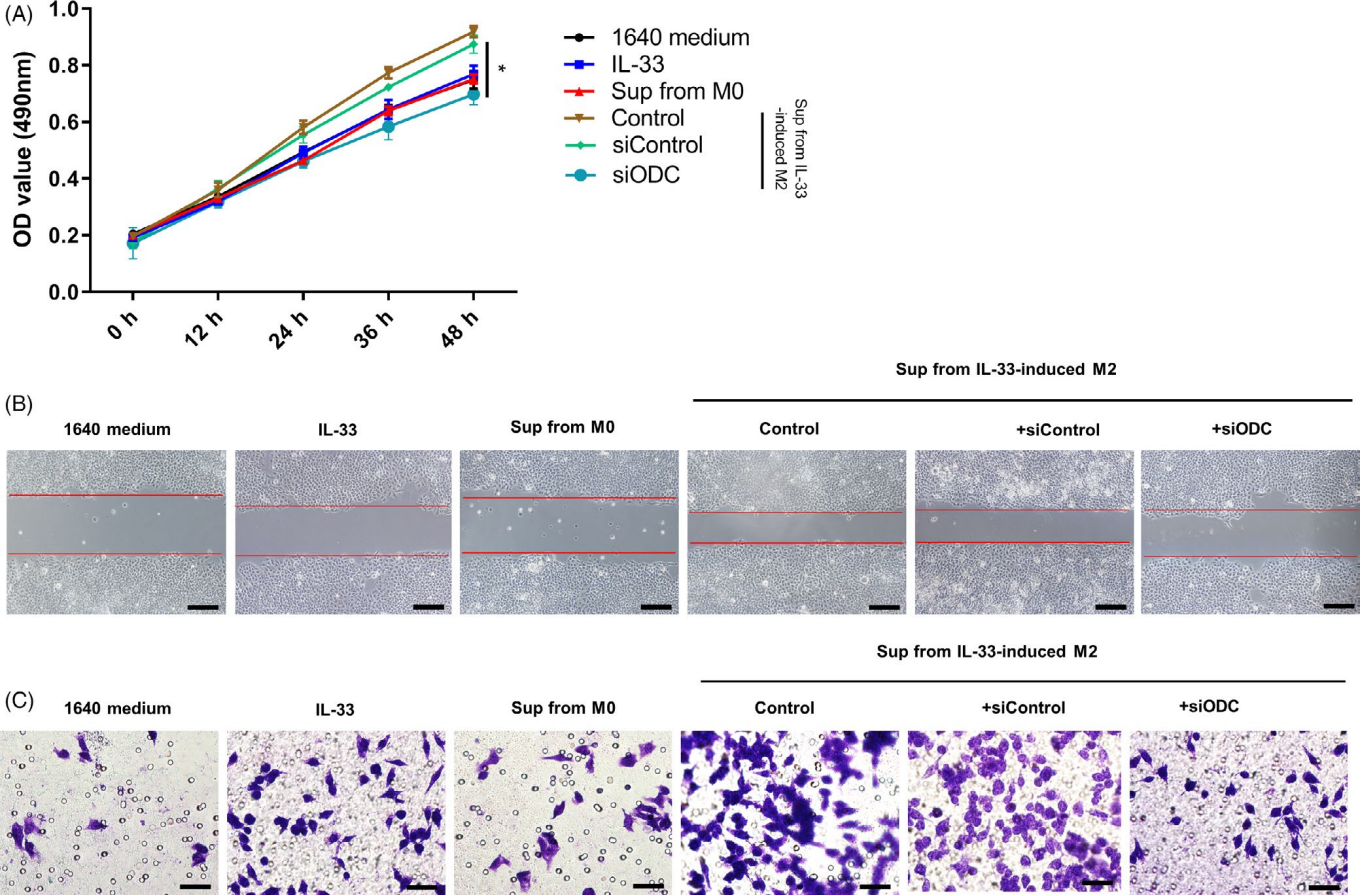
Supernatants from IL‐33–induced M2 macrophages promote ESCC cells proliferation and migration. A, The proliferation of ECA109 cells co‐cultured with the supernatants from M2‐type macrophages with or without knocking down ornithine decarboxylase (ODC), and OD450 values were measured to evaluate the proliferation. OD450 values are presented as the mean ± SEM, n = 5, *P* < .05. B, Migration abilities of the ECA109 cells co‐cultured with supernatants from M2‐type macrophages with or without knocking down ODC were determined by wound‐healing assay and Transwell assay. Representative images were taken at 0 and 48 h in wound‐healing assay (scale bar = 200 μm). C, Representative images of the migration of ECA‐109 cells in Transwell assay and the numbers of migrated cells are presented as the mean ± SEM (scale bar = 50 μm). *P < .05. Bars indicate mean and SEM of triplicate experiments and show a representative experiment of at least 3 independent experiments performed for each panel

We isolated the supernatants from IL‐33–induced M2‐like macrophages and studied their effect on cellular proliferation and migration of the ECA109 cells. We observed that co‐cultivation of ECA109 cells with the IL‐33–induced M2‐like macrophages resulted in a considerable increase in their cellular count compared with the control group (Figure 5A). The statistical count of the cancer cells that permeated the basement membrane was used for evaluating the migration abilities of the ECA109 cells co‐cultured with different supernatants. We found higher cellular migration in the M2 macrophage supernatants group compared with the RPMI‐1640 medium group (Figure 5B‐C). Additionally, siRNA‐induced ODC knockdown attenuated the pro‐migration effect of M2‐like macrophage supernatants (Figure 5B‐C). Thus, these results indicated that IL‐33–induced M2‐like macrophages promoted migration and proliferation of ECA‐109 cells.

### 3.6 ESCC tumour progression was facilitated by IL‐33–induced repolarization of M2‐like macrophages

**Figure 6:**
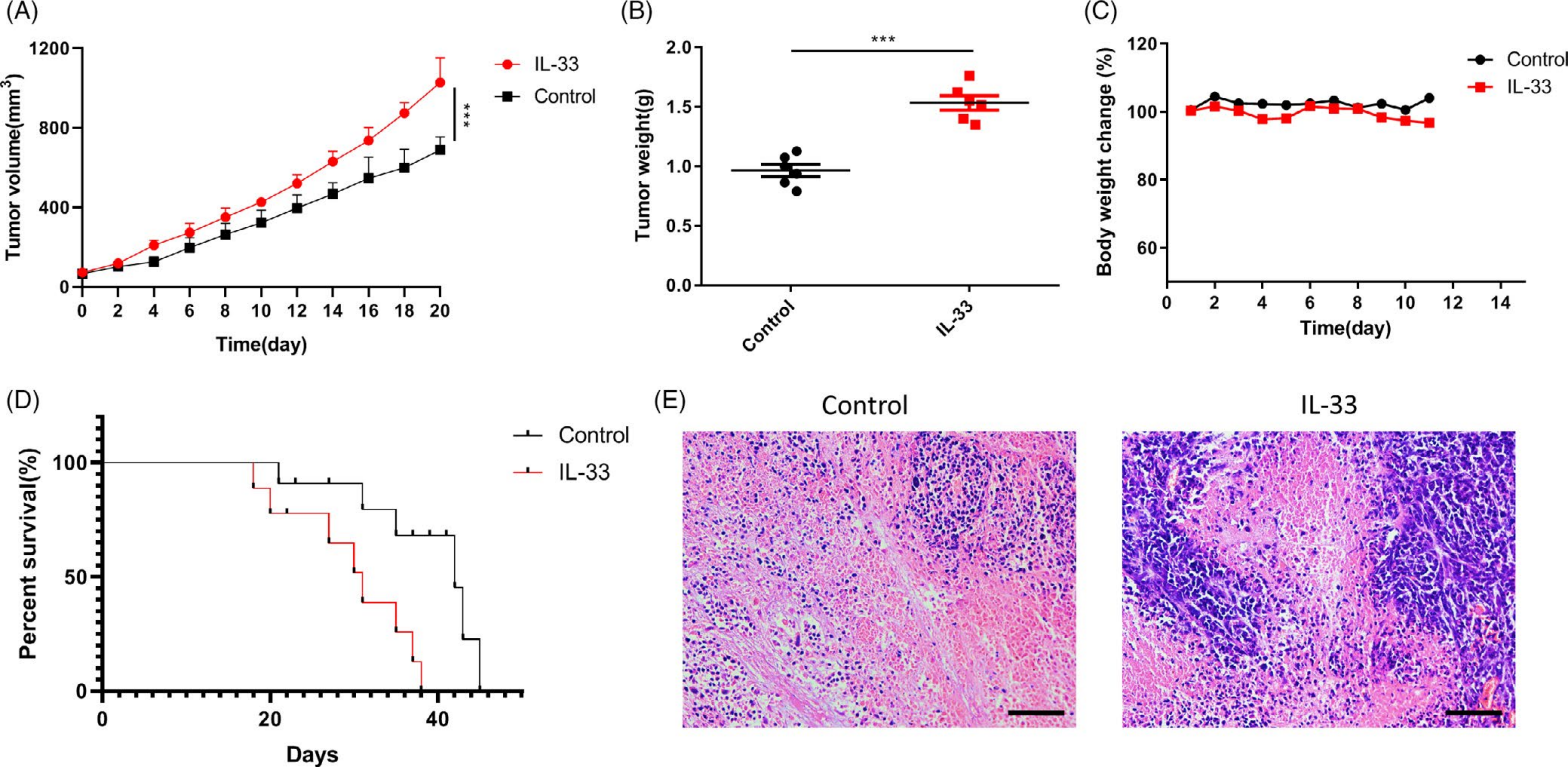
The pro‐tumour effect of IL‐33 in vivo. A, Xenograft tumour growth curve. B, Tumour weight on day 21 after the first administration in each group. C, The body weight change in mice. Results are expressed as mean ± SEM (n = 5; **P < .01, ***P < .001). D, Kaplan‐Meier survival curves of mice received different treatments, n = 10 mice per group. E, H&E staining of tumour tissues (scale bar = 200 μm). ***P < .001. Bars indicate mean and SEM of triplicate experiments and show a representative experiment of at least 3 independent experiments performed for each panel

We subcutaneously injected IL‐33 in the areas surrounding tumour mass every alternate day using PBS as the control. We found that IL‐33 resulted in an enhanced tumour volume compared with PBS (Figure 6A). Similar results were observed for tumour weight (Figure 6B). We did not observe a significant difference in the body weight of mice after the treatment, which indicated the absence of severe side effects due to this treatment (Figure 6C). Additionally, the IL‐33 group had a shorter survival rate compared with the control group (Figure 6D). We observed less necrotic area in the tumour mass of the control group, as evidenced by the H&E staining analysis (Figure 6E).

## 4 DISCUSSION

The TAMs are those macrophages in the tumour microenvironment (TME), which are mainly chemoattracted by monocytes in the blood system by chemokines in TME. The most common chemokines are CSF‐I, CCL‐2, CCL‐3, CCL‐5 and VEGF.^23^, ^24^ M1‐like macrophages participate in the Th1‐type immune response; their differentiation is induced by bacterial products, such as lipopolysaccharide (LPS) or IFN‐γ; they produce toxic intermediates, such as nitric oxide (NO) and reactive oxygen species; they participate in the removal of pathogens and other foreign substances.^5^ M2‐like macrophages participate in the Th2‐type immune response, which is induced by IL‐4, IL‐13 or high expression of immunosuppressive molecules, such as IL‐10, or the high expression of surface molecular markers, such as CD206 and CD163.; they secrete a large number of cytokines, induce angiogenesis and tissue reconstruction, and suppress the immune response of M1‐type macrophages.^6^, ^7^ During the early or non‐progressive stages of malignant tumours, macrophages are recruited into the tumour tissue, to inhibit angiogenesis and activate tumour immunity. As the tumour continues to progress, TAMs are regulated by cytokines in the TME, which is polarized towards the M2 phenotype and promotes tumour development.^8^ In liver cancer, lung cancer and thyroid malignant tumours, high TAM density is strongly correlated with the poor prognosis of patients. A previous meta‐analysis has shown that in more than 80% of the studies, the greater the number of TAMs, the worse the prognosis of patients with malignant tumours.^9^ Current research strategies are using TAMs as a breakthrough for the treatment of tumours. The two main strategies include the reduction of TAMs in the tumours and the conversion of M2‐like macrophages in tumours to the M1 phenotype.^25^

Previous studies have reported the involvement of IL‐33 in the regulation of macrophage polarization. Also, the IL‐13/IL‐4Rα signalling pathway is involved in the amplification and polarization of the alveolar and bone marrow–derived macrophages, thus promoting the development of airway inflammation.^19^ In a murine model, IL‐33 improved CVB3‐induced viral myocarditis by inducing macrophage polarization to the M2 type.^20^

Here, we found elevated levels of IL‐33 in the tumour tissue samples, which were also associated with the upregulated density of CD206^+^ M2‐like macrophages (*R* ^2^ = .65). Thus, we concluded that in ESCC, elevated expression of IL‐33 at the tumour site was correlated with the accumulation of macrophages and M2 macrophage differentiation (Figure 1).

Next, we created an IL‐33–based differentiation system for in vitro stimulation of the macrophages to understand the basic mechanism of IL‐33–induced macrophage differentiation. IL‐10 and TGF‐β are known to be primarily produced by the M2‐type macrophages. Here, we found that the IL‐33–induced macrophages tended towards IL‐10^high^TGF‐β^high^IL‐12p35^low^ phenotypic polarization (Figure 2). ODC gene is the first rate‐limiting enzyme in the process of polyamine synthesis, which has high activity in the tissues with vigorous cell growth and responds quickly to growth‐stimulating factors. Recent studies have found that it plays a very important role in cell proliferation, differentiation, migration, invasion and tumorigenesis and development, and has made some research progress. Previous studies had reported the role of ODC activation in the polarization of M2‐like macrophages in several different types of cancers; however, there are no reports for ESCC. We found that the ARG1 (a biomarker for M2‐like macrophages) and ODC levels in the IL‐33–stimulated macrophages were significantly enhanced (Figure 3). IL‐33 induces ARG1 activity, which converts arginine into ornithine, and ornithine can be decarboxylated by ODC to produce putrescine, which is further converted into spermidine and spermine. The knockdown of ODC considerably decreased the levels of IL‐10, TGF‐β and polyamine (putrescine, spermidine and spermine) levels; however, the IL‐12p35 expression was restored on silencing ODC in the IL‐33–induced M2‐like macrophages, consistent with the previous reports (Figure 4). Thus, the ODC pathway, which promotes M2‐like macrophage differentiation, was involved in the negative regulation of IL‐12p35 expression by IL‐33. These results indicated that the high secretion of IL‐33 induced the infiltration of M2‐like macrophages in ESCC through the ODC pathway.

Additionally, we found a strong correlation between IL‐33 secretion in the ESCC tissue samples and the tumour TNM stages. A significant elevation in cellular migration and proliferation was observed in the ESCC carcinoma cells that were co‐cultured with the supernatant of the M2‐type macrophages in vitro compared with the control group. However, the knockdown of ODC attenuated the M2 supernatant‐induced cellular proliferation and migration (Figure 5). On the contrary, we observed an IL‐33–induced immunosuppressive response in tumour‐bearing mice with no side effects (Figure 6). Thus, IL‐33 secretion in the tumour sites induced M2‐like macrophage differentiation and favoured tumour growth and tumour metastasis in ESCC.

Thus, the results of this study suggest that M2‐like macrophage differentiation is promoted by the elevated secretion of IL‐33 in the tumour sites via ODC activation during the establishment and progression of ESCC, and blocking IL‐33 or ODC may constitute a novel therapeutic route for patients with ESCC.

## CONFLICT OF INTERESTS

The authors declare that there are no conflict of interests.

## AUTHORS' CONTRIBUTIONS

SJM, JJJ and KCC designed the study. SJM, LL, JJJ, PFR and DWD performed the experiments and formal analysis. SJM, JJJ and HFW performed the data curation. SJM and JJJ wrote the manuscript. LL and KCC revised the manuscript and provided professional language editing service. All authors read and approved the final manuscript.

## Data Availability

All data generated or analysed during this study are included in this article.
